# Primary Pancreatic Lymphoma: An Uncommon Presentation in the Pancreatic Tail

**DOI:** 10.7759/cureus.5479

**Published:** 2019-08-25

**Authors:** Yousaf Zafar, Anahat Kaur, Fady Banno, Shrestha Anuj

**Affiliations:** 1 Internal Medicine, University of Missouri - Kansas City School of Medicine, Kansas City, USA; 2 Internal Medicine, University of Missouri - Kansas City School of Medicine, Kansas City, USA; 3 Hematology / Oncology, University of Missouri - Kansas City School of Medicine, Kansas City, USA

**Keywords:** primary pancreatic lymphoma, lymphoma of b-cell origin, pancreatic cancer, pancreatic mass, diffuse large b-cell lymphoma (dlbcl)

## Abstract

Primary pancreatic lymphoma is a rare form of pancreatic cancer that represents a diagnostic and therapeutic challenge due to its rarity and presentation mimicking pancreatic adenocarcinoma. Herein, we report a case of a 57-year-old Caucasian male who presented with left-sided chest pain, epigastric pain, and melena. Abdominal imaging was remarkable for a large, necrotic mass near the tail of the pancreas extending into the splenic hilum and left kidney. Biopsy of the mass confirmed lymphoma of B-cell origin. The patient was diagnosed with Stage IV disease and started on chemotherapy. This case combines an uncommon presentation of lymphoma with a rarely documented primary site in the tail of the pancreas.

## Introduction

Primary pancreatic lymphoma (PPL) is an extremely rare form of pancreatic cancer that often represents a diagnostic and therapeutic challenge due to its rarity and presentation with clinical features that mimic pancreatic adenocarcinoma. PPL constitutes < 0.5% of all pancreatic masses with a histological analysis required for the final diagnosis. It is mostly found in the head of the pancreas; however, it has been reported in other parts of the pancreas as well [1-3].

## Case presentation

A 57-year-old male with a past medical history of hypertension, methamphetamine abuse, and subarachnoid hemorrhage presented to the emergency department with the chief complaint of epigastric abdominal pain, chest pain, melena, fatigue, and light-headedness for several weeks. Epigastric pain was associated with nausea and exacerbated by eating. He denied any previous history of coronary artery disease, pancreatitis, gastric ulcers, and upper or lower gastrointestinal bleed. The patient denied the chronic use of non-steroidal anti-inflammatory medications. He had never had esophagogastroduodenoscopy (EGD) or colonoscopy done in the past.

**Figure 1:**
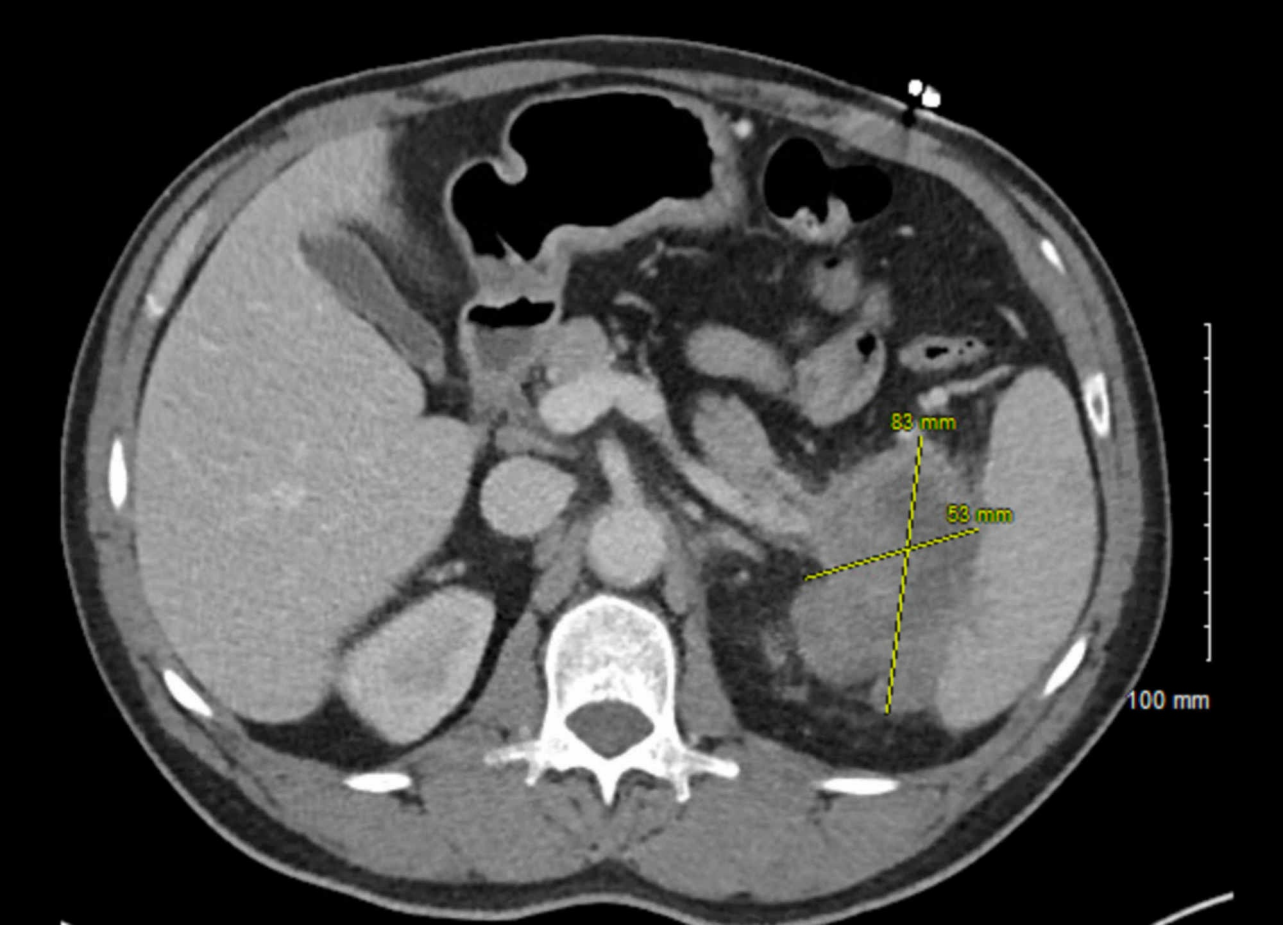
Computed tomography scan of the abdomen showing an 8.3 x 5.3 cm mass centered around the splenic hilum and tail of the pancreas.

On admission, lab work revealed that the patient had anemia with hemoglobin of 8.6 g/dl. White cell count was normal at 7.81 x 103/µL. The patient’s liver function tests, lipase level, and basic metabolic panel results were within normal limits. Troponin-T was negative. His urine toxicology screen was positive for methamphetamines. Imaging studies, including a computed tomography (CT) scan of the abdomen and pelvis and magnetic resonance cholangiopancreatography (MRCP), revealed a large, heterogeneous, peripherally enhancing mass, with central necrosis, measuring 8.3 x 5.3 cm, centered on the tail of the pancreas. The mass was extending into the splenic hilum and invading the left kidney (Figure 1). Retroperitoneal lymphadenopathy and multiple adjacent soft tissue nodules were noted along with the involvement of the left adrenal gland. Carcinoembryonic antigen (CEA), carbohydrate antigen (CA)-19-9, and alpha-fetoprotein (AFP) were all negative.

**Figure 2:**
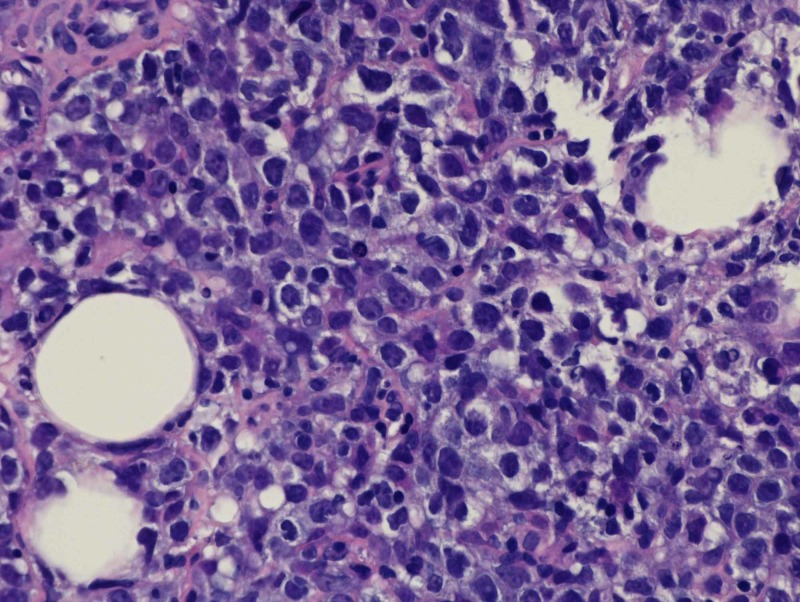
Hematoxylin and Eosin staining at 40x magnification of the core biopsy specimen obtained from the pancreatic mass, showing large cells with vesicular chromatin and small nucleoli. Pathology is consistent with a diffuse large B-cell lymphoma.

A CT-guided core biopsy of the pancreatic mass was undertaken, and this was consistent with lymphoma of B-cell origin (Figure 2).

A positron emission tomography (PET) scan showed increased uptake associated with the large mass centered around the tail of the pancreas and splenic hilum. Also, increased uptake was noted in multiple, additional soft tissue nodules and lymph nodes in the abdomen. No bone marrow involvement was seen on PET scan. A bone marrow biopsy was attempted; however, the specimen was inadequate and this was found to be inconclusive. No evidence of metastatic disease was found on CT chest.

The patient is currently being followed up in the oncology clinic and is three months post-diagnosis. He has completed four cycles of chemotherapy with the dose-adjusted Rituximab, Etoposide, Prednisone, Oncovin, Cyclophosphamide, Hydroxydaunorubicin (REPOCH) regimen and tolerated well other than nausea, vomiting, pancytopenia, and persistent symptomatic anemia requiring intermittent blood transfusions. The PET/CT scan obtained after cycle three showed a significant decrease in size and decreased fluorodeoxyglucose (FDG) affinity of the infiltrative pancreatic tail mass, suggesting marked improvement as compared to the prior study done at diagnosis. Near-complete resolution of retroperitoneal lymphadenopathy was also noted. The plan is to complete a total of six cycles of chemotherapy.

## Discussion

PPL is a rare form of extranodal non-Hodgkin lymphoma (NHL). Approximately 0.2%-2% of patients with NHL have pancreatic involvement at presentation [1-3]. Volmar et al. evaluated pancreatic lesions and reported that 14 (1.3%) cases from 1050 fine-needle aspiration biopsies were pancreatic lymphomas [4]. On a literature review, approximately 150 and 20 cases of PPL were found to have been reported in the English and Chinese medical literature, respectively [5].

PPL diagnosis and treatment is challenging due to its anatomic location and presentation, which could mimic pancreatic adenocarcinoma and other histological subtypes. When the bulk of the disease is localized in the pancreas, a PPL diagnosis can be made. Local or distant lymph node involvement may exist; however, therapy usually is targeted at its location in the pancreas [6-7].

The median age for the presentation of PPL is 57.5 years [8]. The majority of the documented cases of PPL are in the head of the pancreas; however, PPL could also be found in the body and tail regions [9]. It has been reported that over half of PPL patients present with an epigastric mass with a diameter greater than 6 cm; our patient presented with an 8.3 x 5.3 cm pancreatic mass [10]. Lab testing is non-specific for the diagnosis of PPL. Lin et al. found that the serum CA 19-9 level in PPL patients was normal or slightly elevated. This is different from pancreatic adenocarcinoma, where almost 80% of patients have high CA19-9 levels [8].

Endoscopic ultrasound (EUS), CT, and magnetic resonance imaging (MRI) are used to evaluate pancreatic lesions. A finding of tumor in the head of the pancreas without main pancreatic duct dilation and lymphadenopathy below the renal veins points to a diagnosis of PPL. On the other hand, lymphoma is ruled out if calcification or necrosis is present [11]. A PET scan is generally helpful in the staging of lymphoma and the definitive diagnosis is made by histological examination [9].

CT- or EUS-guided fine needle aspiration (FNA) biopsy is the initial approach. If FNA is nondiagnostic, laparoscopy or laparotomy may be performed [12]. The histological subtype is a major prognostic factor in nodal and extra-nodal lymphomas. The most common histological subtype of PPL is diffuse large B-cell lymphoma, accounting for 77% to 80% of all patients [8,13-14]. Follicular lymphoma, Burkitt’s lymphoma, small lymphocytic lymphoma, and T cell lymphoma are other presentations of PPL [14-16].

For our patient, initial findings on imaging studies raised concern for primary pancreatic cancer or metastatic disease extending to the spleen and left kidney. CEA, CA-19-9, and AFP testing were negative, therefore, the likelihood of this being adenocarcinoma of the pancreas or cholangiocarcinoma was low. The final diagnosis of PPL was made after a CT-guided core biopsy of the pancreatic mass.

The anthracycline-based chemotherapy regimen is recommended as the standard treatment for PPL. Chemotherapy combined with radiotherapy is considered a better approach. Behrns et al. reported that the median survival time for only chemotherapy or radiotherapy-treated PPL patients was 13 and 22 months, respectively. They also stated that the time could be improved to 26 months with combined chemoradiotherapy [6]. It is important to note that total pancreatectomy is not recommended for the diagnosis or treatment of PPL, as it doesn't affect survival time or with its associated morbidities [8].

## Conclusions

In conclusion, we presented a rare case of PPL presenting with abdominal pain, weight loss, and obstructive jaundice, diagnosed by a CT-guided pancreatic biopsy. PPL should be considered in the differential diagnosis of pancreatic malignancies, including pancreatic adenocarcinoma, as its management differs from that of other types of pancreatic tumors.
